# Efficacy of Simultaneous Intradermal Vaccination of Swine against Porcine Circovirus 2, Porcine Reproductive and Respiratory Syndrome Virus, *Mycoplasma hyopneumoniae* and *Lawsonia intracellularis*

**DOI:** 10.3390/ani11082225

**Published:** 2021-07-28

**Authors:** Jacquelyn Horsington, Maarten Witvliet, Antonius A. C. Jacobs, Ruud P. A. M. Segers

**Affiliations:** MSD Animal Health, 5830 AA Boxmeer, The Netherlands; maarten.witvliet@merck.com (M.W.); ton.jacobs@merck.com (A.A.C.J.); ruud.segers@merck.com (R.P.A.M.S.)

**Keywords:** swine vaccines, intradermal vaccination

## Abstract

**Simple Summary:**

Porcine circovirus 2, porcine reproductive and respiratory syndrome virus, *Mycoplasma hyopneumoniae* and *Lawsonia intracellularis*, are four highly prevalent, major pathogens of swine for which routine vaccination is common. This study determined whether the intradermal application of vaccines against these four pathogens at the same time and at the same anatomical site had any negative effect on vaccine efficacy. Intradermal vaccination and reduced handling moments offer a more animal-friendly method of vaccination. For all four diseases, the efficacy with the combination of vaccines was equivalent to that with the single vaccines.

**Abstract:**

The combined application of vaccines in swine offers many benefits, including reduced time and labour costs, and improved animal welfare, due to fewer injections and manipulations. This study investigated if simultaneous intradermal vaccinations against porcine circovirus 2, porcine reproductive and respiratory syndrome virus, *Mycoplasma hyopneumoniae*, and *Lawsonia intracellularis*, using a specialised needle-free applicator would confer comparable protection against experimental infection compared to the single vaccines. In all cases, the administration of the vaccines together was as efficacious as the administration of the vaccines alone, significantly reducing clinical signs associated with each of the four pathogens.

## 1. Introduction

With an increasing global demand for pork products and high intensity farming practices, tackling numerous diseases of high economic impact is becoming increasingly important. Porcine circovirus 2 (PCV2), porcine reproductive and respiratory syndrome virus (PRRSV), *Mycoplasma hyopneumoniae* (*M. hyo*), and *Lawsonia intracellularis* (*LI*) are four highly prevalent, major pathogens associated with disease in swine for which routine vaccination is common [1,2,3,4].

PCV2 is involved in a number of syndromes, collectively called Porcine Circovirus Diseases [5,6], and is the main causative agent of the post-weaning multisystemic wasting syndrome. *M. hyo* is a respiratory pathogen and is the main cause of enzootic pneumonia, a chronic disease in pig herds [7,8]. PRRS, caused by PRRSV, is endemic in many countries, with PRRSV1 found predominantly in Europe and PRRSV2 found in North America and Asia. The disease is characterised by abortions, increased mortality in piglets, and respiratory disease in weaners and finishers [9,10]. PCV2, PRRSV, and *M. hyo*, are considered to be the most clinically important pathogens in the Porcine Respiratory Disease Complex, a multifactorial and complex disease characterised by respiratory symptoms and poor growth in growing and finishing pigs [11,12]. *LI* is the causative agent of porcine proliferative enteropathy (PPE), an infectious intestinal disease of pigs also known as ileitis [13]. It has a high prevalence worldwide [14,15] and leads to diarrhoea and poor growth performance in clinically affected animals [16].

While multiple interventions, including biosecurity, diet, stocking density, and management contribute to disease control, vaccines are generally the most effective strategy. With numerous infectious diseases impacting swine health and the productivity of the global swine industry, there is often a requirement for complex and time-consuming vaccination programs. In the absence of a single, mixed vaccine efficacious against all four pathogens listed above, facilitating application with a single administration offers many benefits, such as reduced pig handling and injections, thereby saving time for the farmer or veterinarian and minimising the stress on the animal and farm staff. An additional improvement to vaccination approaches is intradermal and needle-free vaccination, for example using the single or twin-nozzle Intradermal Application of Liquids (IDAL^®^, Henke-Sass Wolf, Germany) device. The twin-nozzle IDAL^®^ 3G Twin (Henke-Sass Wolf, Germany) allows vaccines in two separate vials to be applied simultaneously.

Intradermal vaccination stimulates a protective immune response by targeting antigen-presenting cells in the dermis, in close proximity to skin-draining lymph nodes [17,18]. It also has many welfare benefits, minimising the pain and stress associated with vaccination [19,20]. In addition, the occurrence of needle stick injuries to vaccine administrators and accidental transmission of infections is reduced, and broken needles in the muscle and derived consumer products are prevented. 

In recent years, a number of swine vaccines for intradermal administration have been developed [21,22,23,24]. Porcilis PRRS ID is a live-attenuated PRRSV1 vaccine, which is reconstituted from freeze-dried in the adjuvant Diluvac Forte before application. Porcilis PCV ID is a PCV2 virus-like particle (VLP) vaccine in ready-to-use form in the adjuvant X-Solve12. Porcilis M Hyo ID ONCE is a ready-to-use vaccine comprising inactivated whole *M. hyo* in X-Solve50 adjuvant. Porcilis Lawsonia ID is a freeze-dried vaccine containing inactivated *LI*, which is reconstituted just before use in either X-Solve12 or Porcilis PCV ID.

Porcilis PCV ID and Porcilis M Hyo ID ONCE have been shown to be safe and efficacious when used concurrently, where the vaccines are given at the same time but at different sites on the animal [22]. The efficacy of Porcilis PCV ID in associated mixed use with Porcilis Lawsonia ID has also been reported [21]. Utilising this associated mixed use, all four vaccines can be administered at the same vaccination moment, for example using an IDAL^®^ 3G Twin and IDAL^®^ 3G Mono.

The objective of this study was to test the efficacy of vaccination with Porcilis PCV ID, Porcilis M Hyo ID ONCE, Porcilis Lawsonia ID and Porcilis PRRS ID intradermal vaccines when administered at the same time at the same anatomical site (i.e. on the same side of the neck). Piglets were vaccinated at three weeks of age, and either challenged with PCV2 two weeks post-vaccination (wpv), PRRSV1 4 wpv, *M. hyo* 3 wpv, or *LI* 4 wpv. The comparison between results obtained from the combined-vaccinated and the single-vaccinated animals against each pathogen served as a basis for assessment of interference between the vaccines.

## 2. Materials and Methods

### 2.1. Animals

Progeny (male and female) of several pregnant sows were used for each study. As piglets were vaccinated before weaning and some animals received live PRRS vaccine while others did not, cross-fostering was carried out at 24–48 h of age, ensuring an even distribution of the different litters over the groups.

### 2.2. Vaccines

The vaccines used in the study were Porcilis PCV ID, Porcilis PRRS ID, Porcilis M Hyo ID ONCE, and Porcilis Lawsonia ID. The vaccines were administered intradermally using the IDAL^®^ 3G Twin and IDAL^®^ 3G Mono. The volume of vaccination was 0.2 mL. The dose of each vaccine (when administered singly or in combination) was that which was recommended by the manufacturer. For Porcilis PRRS ID, the dose administered was 1 x 10^4^ TCID_50_ in the PRRSV challenge study and 1 × 10^6^ TCID_50_ in the other studies. Lyophilised Porcilis PRRS ID was reconstituted with Diluvac Forte. Lyophilised Porcilis Lawsonia ID was reconstituted in either Porcilis PCV ID or X-Solve12.

### 2.3. Study Groups

In each study (PCV, PRRS, *M. hyo*, or *LI*), a group of pigs (designated G1 in each study) was vaccinated with Porcilis Lawsonia ID reconstituted with Porcilis PCV ID, and Porcilis M Hyo ID ONCE using an IDAL^®^ 3G Twin, and Porcilis PRRS ID using an IDAL^®^ 3G Mono, administered at the same anatomical site (neck) on the same side of the pig. A second group (designated G2 in each study) was vaccinated with the relevant single vaccine: Porcilis PCV ID, Porcilis PRRS ID, or Porcilis Lawsonia ID. In the *M. hyo* study, this group received both Porcilis M Hyo ID ONCE and Porcilis PRRS ID (on different sides of the pig). A third group (designated G3 in each study) was left unvaccinated (or received only Porcilis PRRS ID in the *M. hyo* study) and served as a challenge control.

### 2.4. PCV Study Design and Sample Analysis

Piglets (breed TN70) negative for PCV DNA and with PCV maternally-derived antibodies <6 log_2_ (in ELISA) were allotted into 3 treatment groups of 15 piglets each. At approximately three weeks old, animals in PCV-G1 were vaccinated intradermally with the vaccine combination and in PCV-G2 with Porcilis PCV ID only. PCV-G3 was not vaccinated and served as a challenge control. At two weeks post-vaccination (5 weeks of age, study day (SD) 14) all animals were challenged with 5.8 log_10_ TCID_50_ of wild-type PCV2b challenge virus, strain I-12/11, applied intranasally. All piglets were observed daily for clinical signs. Blood samples and faecal swabs were collected throughout the study. Three weeks post-challenge, all animals were euthanized by electric stunning followed by exsanguination, and inguinal lymph node; mesenteric lymph node, tonsil, and lung were sampled for the detection of PCV2 nucleic acid. 

Blood samples were allowed to clot, and serum was prepared and stored at −20 °C until tested. Anti-PCV2 antibodies were measured by ELISA, as described previously [25]. 10% tissue sample homogenates were prepared in phosphate-buffered saline. DNA in serum, rectal swabs and tissue homogenates was extracted using a commercial kit (Roche, Magnapure 96 with DNA/viral NA SV kit), and PCV2 DNA was quantified by qPCR, using primers and a probe specific for PCV2-ORF2. The cycle number where specific fluorescence exceeds the threshold is correlated with the cycle numbers for a set of samples containing known amounts of a PCV2-ORF2-containing plasmid. Results were expressed as log_10_ copies/µL of extracted DNA (log_10_ c/µL). Values lower than 1.00 log_10_ c/µL were considered negative and taken as 0.00 log_10_ c/µL for calculation purposes.

### 2.5. PRRSV Study Design and Sample Analysis

Piglets (breed Duroc and York) negative for PRRSV and PRRSV antibodies were allotted into 3 treatment groups of 15 piglets each. At approximately three weeks old, animals in PRRS-G1 were vaccinated intradermally with the vaccine combination and in PRRS-G2 with Porcilis PRRS ID only. PRRS-G3 was not vaccinated and served as a challenge control. At four weeks post-vaccination (7 weeks of age, SD28) all animals were challenged with 5.3 log_10_ TCID_50_ virulent PRRSV Type 1 strain, Isolate 2, applied intranasally.

All piglets were observed daily for clinical signs. Rectal temperatures were taken from one day before challenge to 14 days post-challenge (SD27–42). Pigs were weighed on SD27 and SD56. The average daily weight gain (ADWG) was calculated for each individual animal and averaged by group. Blood samples were collected throughout the study, allowed to clot, and serum was prepared and stored at −20 °C until tested. Anti-PRRSV antibodies were measured by ELISA using a commercial test (IDEXX^®^ PRRS X3), according to the manufacturer’s instructions. With this kit, an S/P ≥ 0.4 is considered positive. PRRSV in serum was quantified by titration on porcine alveolar macrophage (PAM) cells. Briefly, PAMs seeded in 96-well tissue culture plates were inoculated using 25 µL of 10-fold serial dilutions (6 wells per dilution) of the test sample and incubated at 37°C. Cells were assessed for PRRSV infection after 6–7 days by looking for CPE. Titres were calculated using the method of Spearman–Kärber and expressed as log_10_ TCID_50_/mL. All animals were euthanized on SD56 by electric stunning followed by exsanguination.

### 2.6. M. hyo Study Design and Sample Analysis

Piglets (breed TN70) with no or only low antibody titres against *M. hyo* and PRRSV were allotted into 3 treatment groups of 20 piglets each. At approximately three weeks old, animals in Mhyo-G1 were vaccinated intradermally with the vaccine combination and in Mhyo-G2 with just Porcilis M Hyo ID ONCE and Porcilis PRRS ID (using the IDAL^®^ 3G Twin). Mhyo-G3 was vaccinated with Porcilis PRRS ID only and served as a challenge control.

Three weeks post-vaccination, all animals were infected with a virulent Danish *M. hyo* field isolate (provided by Dr. N. Friis, National Veterinary Laboratory, Copenhagen) by intratracheal inoculation on two consecutive days with 10 mL pure culture (10^9^ and 10^8^ colour changing units/mL for the first and second day of challenge, respectively). Blood samples were taken prior to vaccination, just before challenge infection, and at necropsy and serum tested for antibodies using the IDEXX^®^ M hyo Ab test and the IDEXX^®^ PRRS X3 Ab test according to the manufacturer’s instructions. Three weeks post-challenge infection, all animals were euthanised by electric stunning followed by exsanguination and examined for lung lesions. Scoring of the lung lesions was performed according to Ph.Eur monograph 2448.

### 2.7. Lawsonia Study Design and Sample Analysis

Piglets (breed Duroc and York) negative for *LI* and with low maternally-derived antibodies against *LI* were allotted into 3 treatment groups of 25 piglets each. At approximately three weeks old, animals in Laws-G1 were vaccinated intradermally with the vaccine combination and in Laws-G2 with just Porcilis Lawsonia ID. Laws-G3 was not vaccinated and served as a challenge control. At four weeks post-vaccination (7 weeks of age, SD28) all animals were challenged orally with 20 mL homogenized *LI* infected intestinal mucosa.

After challenge, the pigs were observed daily for clinical signs of *LI* infection. Scoring was performed according to Jacobs et al. [21]. In this challenge, model clinical signs become apparent in the third week after challenge. The daily clinical scores 13 to 21 days post-challenge were added up and averaged by group.

The pigs were weighed 1 day before challenge, and on days 6, 13, and 20 after challenge. The ADWG in the third week after challenge (days 13 to 20 post-challenge) was calculated for each individual animal and averaged by group.

Three weeks after challenge (SD49), the pigs were euthanised by electric stunning followed by exsanguination, and a post-mortem examination was carried out. During necropsy the intestines, in particular the ileum (i.e., the distal 50 cm of the small intestine), were examined for lesions indicative of PPE. A faecal sample (from the rectum) and an ileum mucosa sample (5 cm above the ileo-caecal junction) were collected from each animal for testing in a *LI*-specific qPCR. In addition, an ileum sample was collected and fixed in 4% buffered formalin and then further processed into slides. These slides were stained with Haematoxylin–Eosin (HE stain) and with an immunohistochemical (IHC) stain using an anti-*LI* monoclonal antibody (IHC stain) and were examined microscopically. The monoclonal antibody used for the IHC was an in-house developed monoclonal that recognises a surface carbohydrate antigen of *LI*.

The ileum mucosa was macroscopically scored as described in Jacobs et al. [21]. In addition, the percentage of the ileum affected was estimated as follows: the length of the affected part of the ileum was divided by the length of the ileum and multiplied by 100. The total ileum lesion score was calculated by multiplication of the ileum mucosa score and the percentage of ileum affected. The average total ileum lesion score was calculated for each treatment group.

The histological scoring (HE score and the IHC score) was performed as described in Jacobs et al. [21]. The total histological score was calculated by multiplication of the HE score and the IHC score. The average total histological score was calculated for each group. 

Blood samples were allowed to clot, and serum was prepared and stored at −20°C until tested. Serum samples were tested in an in-house *LI* antibody ELISA, as described previously [21]. DNA was extracted from 0.2 g faeces or mucosa sample using a commercial kit (Roche, Magnapure 96 with DNA/viral NA SV kit) and *LI* DNA was quantified by an in-house qPCR, as described previously [21].

### 2.8. Statistical Analysis

Statistical analyses were carried out using SAS (SAS Institute Inc. Cary, NC, USA). Tests were two-sided, using a significance level (alpha) of 5%. 

#### 2.8.1. PCV2

PCV2 titres at time of vaccination were analysed by the Analysis of Variance (ANOVA). PCV2 antibody titres at the time of challenge were analysed by ANOVA with titre at vaccination as the covariate. PCV2 titres after challenge were analysed by ANOVA for repeated measurements. For viral load in serum and rectal swabs, the Area-Under-the-Curve (AUC) of the qPCR results after challenge was calculated by the trapezoidal rule and analysed by the Kruskal–Wallis test. qPCR results in each lymphoid tissue/organ were analysed by the Kruskal–Wallis test.

#### 2.8.2. PRRSV

Body temperature after challenge was statistically analysed by ANCOVA for repeated measurements using the average pre-challenge temperature as the covariate. ADWG was statistically analysed by ANCOVA using the pre-challenge weight as the covariate. PRRSV viremia after challenge was analysed by calculation of the AUC. A virus titre of <1.15 log_10_/ml was set to a titre of 0.00 log_10_/ml in the calculations. The AUC was calculated by means of the linear trapezoidal rule and statistically analysed by ANOVA. Additionally, viremia data was converted into a positive/negative outcome over time for each piglet and statistically analysed by Generalized Estimating Equations (GEE), accounting for the correlation in the repeated measurements on an animal. The p-value was based on the GEE empirical standard error. As part of this, the odds ratio (OR) with its 95% confidence interval was calculated. The OR provides a relative measure of the effect of vaccination in reducing viremia.

#### 2.8.3. M Hyo

For lung lesions, a Kruskal–Wallis test was firstly conducted to compare the three groups. If significant, groups were pairwise compared to the control group by the Wilcoxon test. 

#### 2.8.4. LI

Antibody titres were evaluated using descriptive statistics. The average values were plotted with the standard deviation.

The diarrhoea scores between days 13 and 21 were statistically analysed by a cumulative logit model [26] accounting for the correlation in the repeated measurements using GEE with p-values based on the empirical standard error.

The ADWG in this period (days 13 to 20) was calculated and statistically analysed by ANCOVA using the weight at day 13 as a covariate and using Tukey’s post-hoc test to compare groups.

qPCR data from faeces and ileum mucosa samples were log_10_ transformed (after adding 1 to avoid zeros) and expressed in log_10_ pg DNA/μL. The average values were plotted with the 95% confidence interval where no overlap indicates statistical significance. In addition, the AUC was calculated by the linear trapezoidal rule as a measure of total shedding over time. The AUC of the qPCR data of faeces, the faeces qPCR data on day 21, and the qPCR data of the ileum mucosa on day 21 were statistically analysed by ANOVA using Tukey’s post-hoc test to compare groups.

The macroscopic total ileum lesion score and the total histology score were statistically analysed by a cumulative logit model with p-values based on the Likelihood-Ratio. The odds ratio was defined here as the odds of having lower classes in the vaccine group relative to that in the control group. The mortality was evaluated by a generalised linear mixed model for binomials using a logit link with treatment as a fixed effect.

## 3. Results

### 3.1. Protection against PCV Challenge

None of the pigs had any overt clinical signs at any time during the vaccination period or during the challenge period. One animal in group PCV-G2 was found with paralysis and red and swollen eyelids and was euthanised on SD24 for ethical reasons. No direct cause could be identified. 

**Figure 1:**
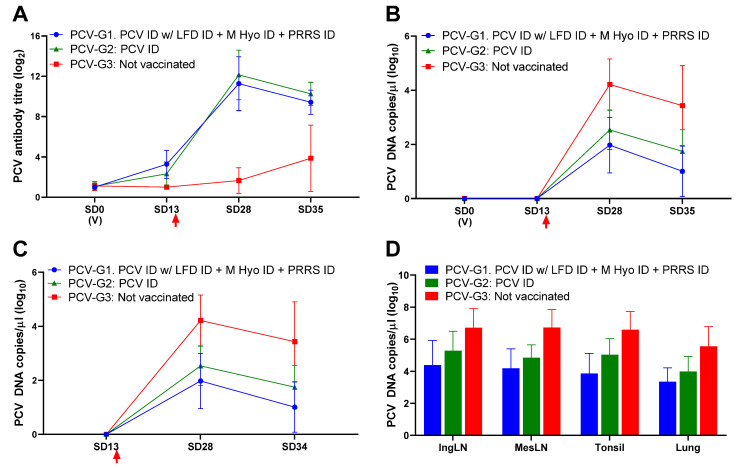
PCV challenge study results. (**A**)—mean antibody response in PCV ELISA; (**B**)—PCV viraemia (mean DNA load in serum); (**C**)—mean PCV DNA load in rectal swab samples; (**D**)—mean PCV DNA load in tissue homogenates. (V) Indicates time of vaccination. Arrow indicates challenge. Error bars show standard deviation.

Pigs receiving the combination vaccination (PCV-G1) had on average higher PCV antibody responses to those receiving Porcilis PCV ID alone 2 weeks post-vaccination (*p* < 0.05), however following challenge, antibody levels were similar between these groups (*p* = 0.4747), but significantly higher than in the control group (both *p* < 0.0001) (Figure 1A). Both PCV-G1 and PCV-G2 showed a significant reduction in viraemia (both *p* < 0.001; Figure 1B), viral load in rectal swabs (both *p* < 0.001; Figure 1C), and viral load in all tissues tested (all *p* < 0.01; Figure 1D), compared to the non-vaccinated controls. Slightly better protection (lower viral loads) was seen in the pigs receiving the combination of vaccines (PCV-G1), however, these differences were not statistically significant. These results indicate that vaccination with Porcilis PCV ID mixed with Porcilis Lawsonia ID, at the same time and anatomical site as Porcilis PRRS ID and Porcilis M Hyo ID ONCE is efficacious against challenge with PCV2. 

### 3.2. Protection against PRRSV Challenge

An error during vaccination resulted in 17 pigs in PRRS-G1 and 13 pigs in PRRS-G2. None of the pigs had any overt clinical signs of PRRS at any time during the vaccination period or during the challenge period and there were no intercurrent deaths during the study. 

**Figure 2:**
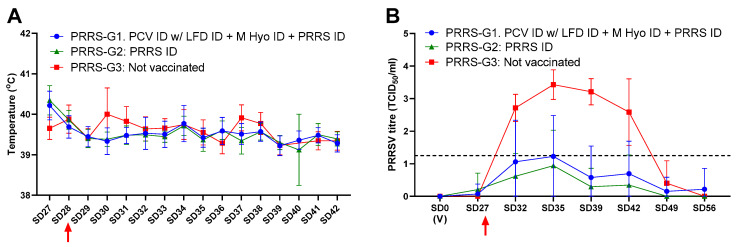
PRRSV challenge study results. (**A**)—mean rectal temperatures during the challenge period; (**B**)—PRRSV viraemia (mean virus titre in serum titrated on PAM cells, dotted line indicates assay cut-off). (V) Indicates time of vaccination. Arrow indicates challenge. Error bars show standard deviation.

On the day of vaccination, all pigs were seronegative for PRRSV. On the day of challenge (SD28), all pigs in the Porcilis PRRS ID group (PRRS-G2) had seroconverted, whereas 87% in the combination group (PRRS-G1) had seroconverted (Table 1). Following challenge with PRRSV1, equivalent protection was seen in PRRS-G1 and G2. An increase in rectal temperature was observed in the non-vaccinated group compared to the vaccinated groups, 2 and 3 days post-challenge (*p* < 0.001), however, no animals were pyrexic (>41.5 °C) during the study (Figure 2A). The vaccinated pigs (PRRS-G1 and G2) had similar ADWG, which, for both groups, was significantly higher than the non-vaccinated pigs (for both *p* < 0.0001; Table 1). Both PRRS-G1 and PRRS-G2 showed a significant reduction in viraemia compared to the non-vaccinated controls (for both *p* < 0.0001; Figure 2B). These results indicate that vaccination with Porcilis PRRS ID, Porcilis M Hyo ID ONCE, and Porcilis PCV ID mixed with Porcilis Lawsonia ID, at the same time and anatomical site, is efficacious against challenge with PRRSV1. 

### 3.3. Protection against M. Hyo Challenge

One pig in Mhyo-G2 could not be transported to the challenge facility because of lameness, and two pigs (Mhyo-G3) were found dead 2 weeks post-challenge. Post-mortem examination identified meningitis as the likely cause of death.

On the day of vaccination, all pigs were seronegative for *M. hyo* and all controls (Mhyo-G3 group) stayed negative until challenge. After challenge, all vaccinated pigs showed a strong anamnestic response, whereas only 5 of the 18 controls had seroconverted at the time of necropsy (data not shown). 

Three weeks post-*M. hyo* challenge, examination of lung lesions showed a significant reduction (*p* < 0.05) in LLS Mhyo-G1 and G2 (50% and an 83% reduction in LLS, respectively) compared to the control group Mhyo-G3 (Table 2). These results indicate that vaccination with Porcilis M Hyo ID ONCE, Porcilis PRRS ID, and Porcilis PCV ID mixed with Porcilis Lawsonia ID, at the same anatomical site, is efficacious against challenge with *M. hyo*. The difference between Mhyo-G1 and Mhyo-G2 was not statistically significant.

### 3.4. Protection against LI Challenge

On SD32, one pig in Laws-G3 was euthanized after days of increasing locomotory and neurological signs (humane endpoint). Necropsy revealed fibrinous polyserositis involving right tarsus, abdominal cavity, and meninges. *Streptococcus suis* was isolated from tarsus and meninges. 

**Figure 3:**
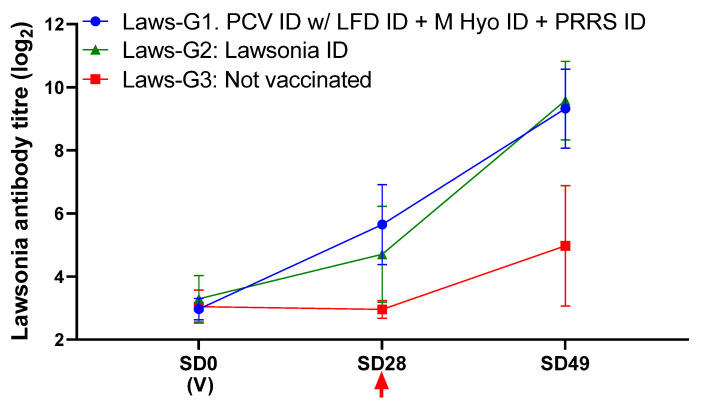
Antibody response to *LI* in ELISA. (V) Indicates time of vaccination. Arrow indicates challenge. Error bars show standard deviation.

Pigs receiving the combination vaccination (Laws-G1) had similar *LI* antibody responses to those receiving Porcilis Lawsonia ID alone (Figure 3). Both Laws-G1 and Law-G2 showed a significant reduction in diarrhoea scores, *LI* DNA load in faeces (both at SD49/21 dpc, and throughout the study, as indicated by AUC) and ileum mucosa, macroscopic ileum score, and microscopic ileum score, compared to the non-vaccinated controls (Table 3). These results indicate that vaccination with Porcilis PCV ID mixed with Porcilis Lawsonia ID at the same anatomical site as Porcilis PRRS ID and Porcilis M Hyo ID ONCE, is efficacious against challenge with *LI*.

## 4. Discussion

The combined application of vaccines in swine offers many benefits, including reduced time and labour costs, and improved animal welfare due to fewer injections and manipulations. The efficacy of multivalent or mixed vaccines against PCV and *M. hyo*, or PCV and *LI*, has been previously reported under both laboratory and field conditions [7,21,24,27]. Therefore, it was of interest to investigate whether vaccination against PCV2, PRRSV, *M. hyo*, and *LI*, at the same time and the same anatomical site using two inactivated, one VLP, and one modified-live virus (MLV) vaccine, would have any negative impacts on vaccine efficacy. Porcilis PRRS ID is an MLV vaccine, and PRRSV is known to dampen the immune response. Infection with PRRSV or use of an MLV PRRSV vaccine was previously reported to reduce the efficacy of *M. hyo* vaccines [28], however, in contrast, no interference was observed with porcine parvovirus vaccine [29,30]. In this study, no significant differences in clinical signs were observed between the groups receiving the combination of vaccines and the groups receiving the single vaccines, regardless of the challenge pathogen, indicating no interference between the vaccines or negative effects on efficacy from the simultaneous administration.

Challenge studies with each of the four pathogens were performed to compare protection from all four ID vaccines administered at the same site on the pig, to protection with the single vaccines (or with PRRSV vaccine in the case of M hyo). In all studies, the vaccines’ safety profiles were similar with either the single or multiple vaccinations, with only mild, transient local reactions (e.g., swelling) at the vaccination sites (data not shown). In the PCV2 challenge, there was a significant reduction in viraemia and viral load in faecal swab samples and tissues in both the combination group and single vaccine group, when compared to the control group. In the PRRSV challenge, there was a significant reduction in viraemia and an increase in ADWG in both the combination group and single vaccine group, when compared to the control group. In the *M. hyo* challenge, there was a significant reduction in the lung lesion scores in both the combination group and single vaccine group, when compared to the control group. In the *LI* challenge, there was a significant reduction in diarrhoea scores, *LI* DNA load in faeces and ileum mucosa, macroscopic ileum scores, and microscopic ileum scores, in both the combination group and single vaccine group, when compared to the control group.

Taken together, these findings support that it is possible to vaccinate intradermally with a needle-free device (two handlings at the same moment) against four major swine diseases with no vaccine interference or negative impact on the efficacy of any of the vaccines used. The IDAL^®^ 3G Twin and IDAL^®^ 3G allow easy and rapid vaccination of pigs with all the added benefits of a needle-free of vaccination, improving convenience and animal well-being. 

In intensive farming situations, multiple factors can contribute to stress in pigs. Routine practices, such as vaccination, involving handling and pain can negatively impact the immune system and lead to reduced health and growth [19,31,32]. Reduced handling and vaccination moments, and intradermal administration of vaccines can not only improve these outcomes but has significant impacts on animal welfare in general [19,20], highlighting the importance of our findings that combined intradermal administration of vaccines against four major swine diseases provides equivalent protection to the administration of the vaccines individually.

## 5. Conclusions

The efficacy of vaccination against PCV2, PRRSV, *M. hyopneumoniae*, and *L. intracellularis* with vaccines administered intradermally at the same time and at the same anatomical location was equivalent to that with the single vaccines, significantly reducing clinical signs associated with each of the four pathogens. The intradermal vaccination and reduced handling and vaccination moments also offer benefits for general animal health and welfare.

## Figures and Tables

**Table 1:** Seropositivity to PRRSV pre-vaccination (SD0), pre-challenge (SD27) and end of study (SD56), and average daily weight gain post-challenge (SD27–SD56).

Group	% Seropositive to PRRSV Antibodies			ADWG (g) [st.dev.] *
	SD0	SD28	SD56	
PRRS-G1: PCV ID/LFD ID + M Hyo ID + PRRS ID	0.0	87.0	100.0	818 ^a^ [±89]
PRRS-G2: Porcilis PRRS	0.0	100.0	100.0	812 ^a^ [±129]
PRRS-G3: Not vaccinated	0.0	0.0	93.0	655 ^b^ [±60]

* ADWG with different superscript (a,b) are significantly different from each other (*p* < 0.0001).

**Table 2:** Median lung lesion scores per group and reductions in the median compared to control group 3.

Group	Treatment	Lung Lesion Score	
		Median *	Reduction
Mhyo-G1	PCV ID/LFD ID + M Hyo ID + PRRS ID	2.6 ^a^	50%
Mhyo-G2	M Hyo ID + PRRS ID	0.9 ^a^	83%
Mhyo-G3	PRRS ID	5.2 ^b^	-

* Scores with different superscript (a,b) are significantly different from each other (*p* < 0.05).

**Table 3:** Post-*LI* challenge results (mean +/− standard deviation).

Group	Mean Diarrhoea Score 13–21 dpc	ADWG g/day 13–20 dpc	qPCR Faeces (Mean log_10_ pg DNA/μL)		qPCR Ileum Mucosa 21 dpc (Mean log_10_ pg DNA/μL)	Mean Macroscopic Ileum Score 21 dpc	Mean Microscopic Ileum Score (IHC) 21 dpc
			AUC	21 dpc			
Laws-G1	0.12 ± 0.44 ^a^	1074 ± 240 ^a^	0.14 ± 0.59 ^a^	0.10 ± 0.34 ^a^	0.04 ± 0.11 ^a^	0.0 ± 0.0 ^a^	0.3 ± 0.9 ^a^
Laws-G2	0.08 ± 0.28 ^a^	1002 ± 303 ^a^	0.69 ± 1.38 ^a^	0.11 ± 0.40 ^a^	0.07 ± 0.21 ^a^	8 ± 26 ^a^	0.7 ± 1.9 ^a^
Laws-G3	2.00 ± 4.34 ^b^	668	2.90 ± 1.75 ^b^	1.18 ± 1.00 ^b^	0.43 ± 0.38 ^b^	109 ± 153 ^b^	4.1 ± 3.4 ^b^

^a^ is significantly different to ^b^ within each category.

## Data Availability

Data can be made available upon reasonable request to the corresponding author.
